# Immunomodulatory functions of fungal melanins in respiratory infections

**DOI:** 10.1128/mbio.02141-25

**Published:** 2025-12-11

**Authors:** Kyle J. Basham, Rebecca A. Ward, Jatin M. Vyas, Kirstine Nolling Jensen

**Affiliations:** 1 Division of Infectious Diseases, Massachusetts General Hospital 2348, Boston, Massachusetts, USA; 2 Vagelos College of Physicians and Surgeons, Columbia University 5798, New York, New York, USA; Vallabhbhai Patel Chest Institute, Delhi, India

**Keywords:** mycology, *Aspergillus*, innate immunty, *Cryptococcus*, lung epithelium, *Rhizopus*

## Abstract

The rate of invasive fungal infections has risen drastically over the last decade and continues to carry devastatingly high mortality rates. Currently, there are no licensed vaccines and limited antifungal agents in clinical trials for fungal-mediated diseases. The limited effectiveness of FDA-approved antifungal medications against invasive fungal infections and the lack of mechanistic understanding of how these infections manifest pose a significant burden on healthcare systems worldwide. Therefore, understanding the molecular details of the host-fungal interactions has never been more urgent. Here, we examine the role of fungal melanin as a virulence factor through its immunomodulatory effects during respiratory infections. Although previous literature on fungal pathogenicity has touched briefly on fungal pigments, they are incomplete in discussing how melanin dysregulates essential functions of the innate immune system. To provide a contemporary perspective, literature on melanized fungal species commonly associated with infections via the respiratory tract has been reviewed to detail holistic mechanisms by which melanin subverts the immune system and manipulates the respiratory epithelium.

## INTRODUCTION

**Fig 1:**
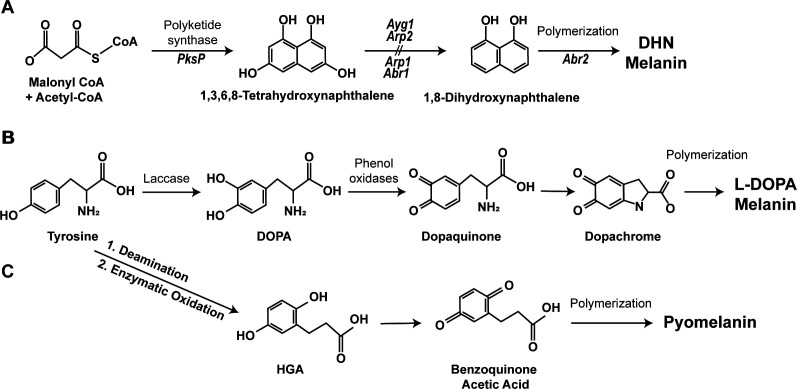
Fungal melanin synthesis pathways for DHN-melanin (**A**), L-DOPA melanin (**B**), and pyomelanin (**C**). The figure is adapted from reference 5.

The fungal cell wall is a distinct entity for human pathogens that protects underlying carbohydrates readily recognized by immune cells, enabling fungi to thwart host immunity and establish disease. Thus, components of the cell wall and enzymes that mediate their formation are targets in the antifungal therapeutic pipeline. The cell wall undergoes remarkable remodeling during infection to shed the outer layers (e.g., melanin, rodlet, and capsule) to establish infection. Melanins, a common cell wall component in pathogenic fungi, are an evolutionarily conserved class of biopolymers ubiquitously expressed in humans, animals, plants, bacteria, and fungi that contribute to pigmentation (1), and the topology of melanin within the fungal cell wall has been illustrated by others (2, 3). Melanins protect against environmental stressors, including enzymatic degradation, radiation, heavy metals, and extreme temperatures (4). In fungi, melanin is mainly produced through three biosynthetic pathways, giving rise to distinct melanin types: 1,8-dihydroxynaphthalene melanin (DHN-melanin), L-3,4-dihydroxyphenylalanine (L-DOPA melanin), and pyomelanin (Fig. 1) (5, 6). Whether these fungal melanins behave simply as a protective layer or participate as a bioactive compound remains an area of interest. Supporting the hypothesized role of protection, melanins permit fungal organisms to survive in virtually inhospitable environments such as high levels of radioactivity at the Chernobyl nuclear power plant or high levels of the unfiltered UV rays in the vacuum of space (7–9). However, emerging evidence reveals an active role in the pathogenesis of fungal melanin as a virulence factor. The number of individuals at risk of severe fungal infections continues to rise, along with elevated mortality rates, highlighting the critical need to understand these fungal melanins better. In this review, we will explore the current knowledge of fungal melanin as a key contributor to pathogenesis and interactions with the airway epithelium and innate immune cells for the major melanized fungal pathogens that cause human respiratory disease.

## MELANIZED FUNGAL PATHOGENS OF THE RESPIRATORY SYSTEM

The annual incidence of invasive fungal infections is estimated to be 6.5 million, of which melanized fungal species, including *Aspergillus* spp., *Cryptococcus* spp., and *Rhizopus* spp., account for an overwhelming portion of this population (10). Despite the presence of melanin in other fungi (e.g.*, Pneumocystis jiroveci*, *Coccidioides* spp., *Histoplasma capsulatum*, *Candida auris*, *Candida albicans*, and *Blastomyces dermatitidis*) (11–14), we will focus on *Aspergillus* spp., *Cryptococcus* spp., and *Rhizopus* spp. as they are highly melanized. While *C. albicans* is considered a WHO high-priority pathogen, much less is known about its melanin production and the impact of melanin on host responses.

### *Aspergillus* spp.

*Aspergillus* spp. remain the most prevalent pulmonary fungal pathogen, causing a spectrum of diseases, ranging from allergic responses to invasive infections (15, 16). The population at higher risk of developing severe *Aspergillus*-related disease continues to rise with the introduction of immunomodulatory therapeutics for autoimmune diseases, cancers, and solid organ transplantation, incidence of pre-existing lung disease, and superinfection following viral infection (i.e., influenza and SARS-CoV-2). A growing number of strains possesses multidrug resistance, which contributes further to the elevated mortality rates and severe disease (17, 18). The current clinical landscape of *Aspergillus*-related diseases highlights the critical need to understand mechanistically how this organism thwarts the host immune system. The presence of melanin on the outer conidial layer beneath a rodlet layer of *Aspergillus* spp. conidia endows the organism to circumvent host immunity. Indeed, infections with amelanotic mutants of *Aspergillus fumigatus* result in reduced pathogenesis and improved host response (19–23). *A. fumigatus* produces DHN-melanin, the dominant melanin form in *Aspergillus* spp., through the catalysis of precursors malonyl-CoA and acetyl-CoA into tetrahydroxynaphthalene by a polyketide synthase and a subsequent series of reactions (Fig. 1A) (24). A cluster of genes, including *pksP* (also referred to as *alb1*), *arp1*, *arp2*, *abr1*, *abr2*, and *ayg1*, is responsible for the biosynthesis of DHN-melanin (25). This DHN-melanin gene cluster is an evolutionarily conserved molecular mechanism of pigmentation in various melanized fungi (26).

### *Rhizopus* spp.

Mucormycosis is the second most common opportunistic mold infection, after aspergillosis, with mortality rates ranging from 38% to 82% (27). Patients with poorly controlled diabetes with associated ketosis, those who are immunologically fragile (hematopoietic stem cell or organ transplantation and oncological patients), trauma or burn victims, or those following SARS-CoV-2 infection are at increased risk (28). Mucormycosis is caused by fungal pathogens in the Mucorales order, most frequently *Rhizopus* spp., *Mucor* spp., and *Lichtheimia* spp. Of these common contributors to mucormycosis, only *Rhizopus* melanin has been studied, although less is known compared to *Aspergillus* and *Cryptococcus* spp. Despite the limited knowledge, *Rhizopus* spp. lacking melanin also display a greater susceptibility to host antifungal activity, indicating that melanin is a virulence factor (29–31). *Rhizopus* produces L-DOPA melanin through a copper-dependent tyrosinase/laccase biosynthetic pathway (Fig. 1B) (29), though the complete annotation of the enzymes used in this pathway is not known.

### *Cryptococcus* spp.

Pulmonary cryptococcosis, caused by the pathogenic yeast *Cryptococcus neoformans* or *Cryptococcus gattii*, occurs primarily in individuals with HIV, diabetes, or malignancies as well as those undergoing organ transplantation and/or receiving immunosuppressive therapies, resulting in mortality rates between 20% and 55% (32). In addition, *C. gattii* has the capacity to cause systemic disease in apparently immunocompetent individuals and those with anti-GM-CSF autoantibodies (33). *Cryptococcus* has an uncanny ability to disseminate, particularly to the central nervous system, often leading to meningoencephalitis (34). Despite differences in the type of melanin compared to *Aspergillus* spp. (i.e., L-DOPA vs DHN)*, Cryptococcus* spp. melanin yields similar suppression of host antifungal responses as *Aspergillus* spp. Amelanotic spores or media-grown spores lacking L-DOPA result in more robust antifungal responses, such as increased susceptibility to macrophage phagocytosis, fungal killing, microbicidal peptides, and oxidative stress (35–37). Furthermore, melanized *Cryptococcus* demonstrates protection from mechanical and environmental stress compared to non-melanized spores (38). The L-DOPA synthesis pathway closely mimics mammalian eumelanin biosynthesis and involves the conversion of precursor molecules, L-dopamine or L-tyrosine, through tyrosinase or laccase enzymatic activity, respectively (Fig. 1B) (24). *C. neoformans* produces L-DOPA melanin through laccase enzymatic activity, which subsequently catalyzes the oxidation of L-DOPA into dopaquinone. Uniquely, an imbalance of the enzymes utilized in this process causes the synthesis of other hydroxylated aromatic compounds, such as homogentisic acid. Interestingly, this acid is believed to influence a separate synthesis pathway required for the formation of pyomelanin (5). Similar to *Rhizopus*, the identification of the enzymes in this pathway is lacking.

## RESPIRATORY EPITHELIUM AND FUNGAL MELANIN

The airway epithelium serves as the first barrier and sensor of inhaled microorganisms, including the melanized fungi *Aspergillus* spp., *Cryptococcus* spp., and *Rhizopus* spp., and coordinates activation of the immune system. Using single-cell RNA-seq, we know that the human airway epithelium is a highly complex tissue with highly specialized cells (39, 40). These cells are composed of common (basal, ciliated, club, and goblet) and rare (ionocytes, neuroendocrine, and tuft cells) epithelial cell types (41, 42). Aside from their well-known protective barrier functions and management of fluid balance, they constitute an essential component of host immunity by clearing pathogens and environmental contaminants through mucociliary movements and orchestrating innate and adaptive immune functions (41, 43).

The first actions in the pulmonary lumen are epithelial-driven mucociliary movements and promoting resident immune cell fungicidal and phagocytic functions (41). Further disrupting the pathogenicity of inhaled organisms, epithelial cells produce antimicrobial proteins and peptides, such as β-defensin, lysozymes, and ferrins, which are present in the mucus layer (44–46). Although most inhaled conidia are cleared in healthy lungs, individuals with structural lung disease carry a heightened infectious risk (47). This lack of clearance causes the fungi to remain viable and initiate their pathogenic mechanism, such as conidial swelling and germination into hyphae. These changes expose fungal cell wall components, commonly referred to as pathogen-associated molecular patterns (PAMPs), which can be recognized by host epithelium and immune cells through pattern recognition receptors (PRRs) to orchestrate innate immune responses (48, 49). Through these interactions, epithelial cells secrete chemotactic molecules and pro-inflammatory mediators (e.g., chemokines and cytokines) to coordinate the innate immune response.

Despite the ability to coordinate host responses, there is increasing evidence that fungal melanin thwarts these antifungal immune responses. Interestingly, wild-type (WT) *A. fumigatus* suppresses epithelial inflammatory responses and ablates transepithelial neutrophil migration (23, 50). However, rodlet- or DHN-melanin-deficient *A. fumigatus* induces swift neutrophil migration across the epithelial barrier (23). This phenomenon is largely caused by a melanin-dependent block of airway epithelial secretion of CXCL1 and CXCL8 in a transcriptional- and translational-independent manner (50). Furthermore, fungal melanin revealed potent scavenger functions of epithelial-derived CXCL10 and CCL20, further preventing an effective immune response (51). Simultaneously, melanin ablates calcium fluxing in airway epithelium and dysregulates actin filamentation in airway epithelium (50). Thus, melanin actively modulates epithelial host responses required to mount an adequate inflammatory response. These findings highlight the importance of elucidating how fungal melanin affects the silencing of airway epithelial cell secretory pathways and subsequent inflammatory responses required for fungal clearance (similar to the mute button on a TV remote control). Interestingly, the CARD9-MyD88 signaling axis may regulate epithelial responses to pathogenic stimuli through the production and secretion of chemokines to further orchestrate inflammatory responses (52). However, the role of fungal melanin on CARD9-MyD88 signaling remains unexplored.

## INNATE IMMUNE CELLS AND FUNGAL MELANIN

Coordination between airway epithelium, resident immune cells (i.e., alveolar macrophages), and circulating immune cells (e.g., macrophages, monocytes, neutrophils, and dendritic cells) is critical to mounting an effective defense against invading pathogens. As first responders to invading pathogens, innate immune cells exert antifungal activity through a myriad of effector functions, including reactive oxygen species (ROS) production, phagocytosis, and secretion of pro-inflammatory mediators. Many of these functions rely on PRRs' interactions with fungal PAMPs or chemokines released by other host cells (53, 54). Ultimately, innate immune cells are responsible for killing invading fungi.

Innate immune cells use autophagy and apoptotic pathways to eliminate pathogens. Autophagy is a highly conserved mechanism in eukaryotes that utilizes lysosome-mediated catabolic processes to degrade and remove dysfunctional intracellular components (55). This mechanism is utilized by specific innate immune cells such as macrophages, neutrophils, and monocytes, therefore playing a major role in the elimination of intracellular pathogens (56). LC3-associated phagocytosis (LAP) is a specialized form of autophagy regulated by the activation of NADPH-mediated ROS production (57). Macrophage using apoptosis as a means for eliminating pathogens was recently reviewed (58). One of the major signaling transduction pathways that mediates survival in immune cells, such as monocytes, macrophages, and DCs, is phosphatidylinositol 3-kinase (PI3K) and its downstream signaling molecule serine/threonine kinase, Akt (59). Despite these key pathways, fungal pathogens have evolved mechanisms to circumvent fungal killing by host immune cells.

### Melanin’s impact on autophagy

Melanin plays a role in the prevention of LAP-mediated fungal killing in macrophages. Prior studies unveiled that removal of *A. fumigatus* melanin was required to activate LAP through exposure of β-glucan during germination via the dectin-1/SYK kinase/NADPH signaling cascade (60, 61). Additionally, melanin from multiple sources (*A. fumigatus*, *Aspergillus nidulans*, and purified melanin) blocked LAP in a NADPH oxidase-dependent manner by selectively excluding the p22*phox* subunit, a critical intermediate of NADPH oxidase, from the phagosomal membrane (61). The activation of NADPH oxidase via the p22*phox* subunit is also regulated by the autophagy protein Rubicon (RUN domain Beclin-1 interaction with the cysteine-rich containing protein) (62). Kyrmizi et al. discovered that fungal melanin inhibits this Rubicon-mediated activation of NADPH oxidase (63). Additionally, they revealed that calcium/calmodulin (CaM) signaling, which regulates LAP, depends on the release of calcium from the phagosomal lumen to the peri-phagosomal area. Therefore, fungal melanin’s ability to block peri-phagosomal calcium release, and in turn, the impairment of calcium effector protein recruitment to the phagosome through the sequestration of calcium inside the phagosomal lumen is the predominant mechanism of calcium/CaM-dependent inhibition of LAP (63).

Interestingly, the inhibition of LAP is a general mechanism to subvert immunity by melanized fungi. Although *Rhizopus* spp. are unable to undergo intracellular conidial swelling in macrophages, *Rhizopus* conidia evade macrophage killing through a melanin-induced inhibition of LAP (29). Although there have been a variety of studies that reported a role of *C. neoformans* in autophagy through characterization of various ATG proteins (64), melanin has not been the focus of these investigations and is consequently outside the scope of this review. However, these observations require further elucidation.

### Melanin’s reprogramming of macrophages

Fungal pathogens evade host defense mechanisms through reduced detection and reprogramming of host immune cells. Unlike melanin-deficient *A. fumigatus*, phagocytosed WT *A. fumigatus* activates and sustains the PI3K/Akt signaling cascade in macrophages, indicating that melanin can alter macrophage programming and function (65). This signaling pathway inhibits apoptotic responses of macrophages and promotes classical macrophage activation, ultimately enhancing conidial survival in host cells (65–67). These results were consistent in other melanized *Aspergillus* spp. and isolated melanins of various types (65).

The Warburg effect, discovered by Otto Warburg in the early 1900s, is characterized by the preference to undergo glucose metabolism through glycolysis rather than oxidizing mechanisms in the mitochondria, even in the presence of oxygen (68). Monocytes, macrophages, dendritic cells, and T lymphocytes are reported to generate a readily available source of energy in this manner. The serine/threonine protein kinase mammalian target of rapamycin (mTOR) and the transcription factor, hypoxia-inducible factor-1α (HIF-1α) regulate this paradoxical phenomenon (69). Across fungi, macrophage Warburg reprogramming appears species specific: *C. albicans* can induce aerobic glycolysis via β-glucan-dectin-1-Akt/mTOR-HIF-1α signaling (70), whereas *A. fumigatus* prominently employs DHN-melanin to drive glycolytic shift (71). By contrast, a melanin-driven Warburg program has not been shown for *C. neoformans*, while lung infection with *C. gattii* has been associated with glycolytic activation and lactate accumulation (72). As mentioned above, intracellular melanin is removed during *A. fumigatus* conidial germination, and the PAMPs in the fungal cell wall are consequently exposed. This shedding of *A. fumigatus* melanin within macrophages activated glycolysis via induction of mTOR-HIF-1α signaling (71). Thus, fungal melanin may modulate macrophage immunometabolism to increase glycolytic processes. In this study, melanin-mediated activation of glycolysis, which is regulated by calcium stores of the endoplasmic reticulum, was independent of extracellular calcium entry (71). Additionally, activation of the SYK kinase complex in the LAPosome simultaneously activates HIF-1α, which upregulates glycolysis and mitochondrial respiration and is inversely regulated to LC3-II (73). Interestingly, HIF-1α also promotes PI3K signaling to propagate classical pro-inflammatory functions and cytokine production by macrophages, thereby driving immune cell infiltration and acute inflammation (74). Further investigation is warranted as this extensive crosstalk between autophagy and macrophage reprogramming could be a fundamental mechanism of pathogenesis for melanized fungal species. The ability of melanin to polarize macrophage metabolism toward glycolysis suggests a potentially interesting therapeutic strategy in targeting glycolysis pathways. Furthermore, melanin-mediated metabolic reprogramming of macrophages and inhibition of autophagy via calcium sequestration requires molecular characterization to illuminate novel pathways for therapeutic intervention.

Iron and other trace minerals are critical for survival in the host. These minerals are critical to protection against oxidative stress, cell growth, and oxygen transport. However, illnesses that require iron transfusions or that lead to an overproduction of iron can leave the host susceptible to infection (75). As a solution, host cells sequester minerals (i.e., iron, zinc, and manganese) to essentially starve pathogens of trace minerals and prevent further pathogenesis during infections, a process called “nutritional immunity” (75). The process of mineral starvation through sequestration is mediated by immune cells, such as macrophages and neutrophils, as well as mucosal epithelial cells in the intestinal tract (75, 76). Some pathogens use serum iron as a mechanism of their pathogenicity. For example, the iron transporter in *Rhizopus oryzae* encoded by *FTR1* enables iron uptake and increases virulence (77). However, alveolar macrophages phagocytose *Rhizopus* spp. and combat this iron-dependent pathogenic mechanism through the sequestration of iron inside the phagosome, in turn leading to fungal growth inhibition (29). In the same study, cell wall melanin in *Rhizopus* conidia halted LAP-mediated phagosome maturation in parallel with the iron sequestration. Whether melanin from other melanized fungi similarly regulates the utilization of trace minerals for pathogenesis to circumvent host-mediated starvation remains an important question for future studies. Moreover, a more detailed explanation must be provided regarding the host immune response in terms of nutritional immunity, as these pathways could possibly lead to future therapeutics to combat fungal infections.

Altogether, melanin from various fungal species displays a general mechanism of virulence in which macrophages are targeted. Even the use of synthetic solubilized L-DOPA melanin inhibited macrophage function by suppressing phagocytosis as well as cytokine (TNFα, IL-1β, and IL-6) and ROS production (78). Our understanding of how L-DOPA melanin in *C. neoformans* shapes host responses remains limited. It remains unclear as to what mammalian cellular receptor(s) recognize fungal melanins. Despite this limitation, data about *Aspergillus* spp. and *Rhizopus* spp. suggest that fungal melanin exploits host machinery through macrophage reprogramming to inhibit their immune function. This maneuver, in turn, leads to more severe pathogenesis, which can effectively burden the host immune response and lead to potentially fatal outcomes.

## THE IMPACT OF FUNGAL MELANINS ON HOST SOLUBLE MEDIATORS

The crosstalk between immune cells is imperative for effective strategies for fungal clearance. Soluble mediators coordinate various immune responses, including the regulation of immune cell proliferation, release of enzymes and acute phase proteins, secretion of immunoglobulins, and cytotoxic immune cell activation. These soluble mediators are critical in the clearance of fungal infections via direct or indirect actions (79). Here, we describe the impact of fungal melanin on soluble mediators, including cytokines, calcium, components of the complement system, surfactant, and oxidative stress mediators.

### Melanin and cytokines

Cytokines are ubiquitous soluble mediators that behave as pivotal signaling molecules to shape the immune responses, such as inflammation, cell signaling, and tissue repair. A subset of cytokines includes “chemotactic cytokines” (i.e., chemokines) that are imperative for leukocyte recruitment to sites of infection or injury. While the intricacies of these mediators fall outside the scope of this review, this subject has been extensively discussed elsewhere (80, 81). With the important role of these mediators, it begs the question: can fungal melanin influence the inflammatory signature of cytokines and chemokines? If so, does melanin directly bind these soluble mediators and/or use indirect action to shape host responses?

DHN-melanin modulates the host immune response through inhibition of PAMP-induced production of cytokines, including TNFα, IL-10, and IL-6 (82). Furthermore, *A. fumigatus* melanin can deplete chemokines from the inflammatory microenvironment by binding and removing CXCL10 and CCL20 from the extracellular space in a transcription-independent manner (51). We have shown that melanin blocks airway epithelial secretion of chemokines CXCL1 and CXCL8, but not CXCL10 or CCL20, in airway epithelial cells, independent of transcription and translation (50). These data indicate that melanin exerts its impact through both direct binding and indirectly, depending on the chemokine. Inhibition of cytokine and chemokine secretion by melanin leads to immunomodulation of host responses and subsequently increases the pathogenesis of *A. fumigatus*.

Although the effects of melanin on host chemokines in the mold species *Rhizopus* have not yet been reported, melanin produced by *C. neoformans* also attenuates host immunity and inflammatory responses through cytokine modulation. Indeed, the supernatant from lung homogenates revealed elevated IL-4, IL-12, and MCP-1 (CCL2) production 7 days post-infection with melanized *C. neoformans* compared to non-melanized cells in tandem with a higher fungal burden (83). Furthermore, this study unveiled an increase in TNFα in a laccase-deficient mutant. Furthermore, L-DOPA-derived synthetic melanin disrupted cytokine production in human monocytes through a post-transcriptional-mediated suppression of TNFα, IL-1β, and IL-6 (84).

These studies suggest that L-DOPA melanin in *Cryptococcus* skews the host response toward a type 2 response. Although the cytokines IL-2, IL-10, and IFN-γ displayed no changes in these infections, the authors did not examine chemokines previously shown to be blunted in DHN-melanin (e.g., CXCL1, CXCL2, CXCL10, and CCL20). A useful tool to study fungal melanin is the production of melanin ghosts. By exposing melanized fungal cells to harsh conditions, which dissolve the internal cell components, this process leaves the melanin shell intact and preserves the original cell’s shape. Our prior work using *C. neoformans* purified melanin ghosts or synthetic L-DOPA in parallel with *Pseudomonas aeruginosa* (used to stimulate high production of chemokines in airway epithelium) drastically suppressed CXCL8 production (50). Although these findings suggest that L-DOPA and DHN-melanin may modulate host immunity in similar manners, whether the effects on chemokines remain the same in melanized versus non-melanized whole organisms warrants further investigation. Additionally, the precise mechanism by which L-DOPA melanin modulates these cytokines and chemokines remains elusive. It is possible that these occur through other soluble mediators, possibly through dysregulation of the complement system or calcium signaling.

### Melanin and calcium

Calcium serves as a ubiquitous second messenger and regulates key host immunity pathways in innate immune cells and epithelial cells, such as cytokine induction, PRRs signaling, and ROS production (85, 86). Previous studies demonstrate the role of fungal melanins in changing host calcium signaling, resulting in increased virulence. Indeed, calcium ions were elevated in the phagolysosomes of macrophages exposed to amelanotic *A. fumigatus* conidia when compared to melanized conidia (87). Furthermore, the reduction in CXCL1 and CXCL8 secretion in epithelial cells is associated with dampened calcium fluxing (50). These data are supported by studies showing that calcium signaling is required for pathogen uptake and CXCL8 secretion in host cells (88, 89). Like *A. fumigatus*, sequestration of calcium was recently reported in *C. neoformans* as melanin’s calcium-binding properties prevented its calcium-dependent capsule growth (90). The question of whether the *C. neoformans* melanin-mediated sequestration of calcium plays a similar inhibitory role in autophagy, like *A. fumigatus* and *Rhizopus*, remains unknown and requires further investigation.

### Melanin and complement

The complement system is a vital component of host defense and inflammation that bridges the gap between the innate and adaptive immunity (91). This complex network consists of more than 50 proteins found as soluble proteins in plasma or as membrane-associated proteins on cell surfaces (92, 93). Activation of the complement system plays a major role in pathogen clearance through opsonin-mediated phagocytosis and cell lysis. The complement system can be activated through three different pathways: classical, lectin, and alternative. The classical pathway is triggered by C1q recognition of antigen-antibody complexes or pentraxins. The lectin pathway uses mannose-binding lectin or ficolin to detect the presence of carbohydrates on pathogens or immunoglobulins. Finally, the alternative pathway becomes activated following hydrolysis of C3. Although the trigger for these three pathways remains distinct, they all converge on the C3 and C5 by the formation of C3/C5 convertases, which activate C5b-9 to induce lytic killing through pore formation on the surface of pathogens.

Melanin shields fungal pathogens from binding with complement components to circumvent effective clearance. Specifically, amelanotic *A. fumigatus* conidia showed elevated C3 binding affinity compared to WT conidia (21, 94). This higher C3 binding further resulted in greater phagocytosis in neutrophils and was associated with improved host survival (21). Given the binding of C3, these data suggest that DHN-melanin blunts the complement system and subsequent opsonization through the alternative pathway. The activation of the complement system, specifically through the alternative pathway, could be a general property of fungal melanin. Indeed, both *Aspergillus niger* and *C. neoformans* (whole organism and melanin ghosts) can bind to C3 and activate the complement system (95). While the kinetics and total deposition of C3 fragments were unaltered by *C. neoformans* melanin ghosts, it is still unclear if this is consistent in melanized versus non-melanized *A. niger*. Furthermore, various pathogenic agents of mucormycosis (i.e., *Rhizopus arrhizus, Rhizopus microsporus*, *Lichtheimia ramosa*, *Lichtheimia corymbifera*, *Rhizomucor pusillus*, and *Mucor circinelloides*) show binding patterns of human C1q, which is indicative of initiation through the classical pathway, C3c, and terminal complex C5b-9 from spores that suggests participation of the complement system (96). Interestingly, *Rhizopus* species revealed lower binding affinities than the other mucormycoses examined, which were associated with lower survival after intravenous infection in a mouse model of infection. One potential explanation for these differences may be due to the fungi-specific melanin content. However, further investigation into whether melanin impacts this activation remains to be elucidated.

### Melanin and surfactant

Surfactant proteins (SPs) are produced by mucosal epithelial cells located in the lungs, gastrointestinal tract, and genitourinary tract, as well as endothelial cells in the heart, brain, and skin (97). In the lungs, alveolar type II cells (also referred to as type II pneumocytes) are the main producers of pulmonary surfactant synthesis and reside in the alveoli, although club cells (formerly known as Clara cells) in the bronchioles also produce these soluble mediators (98). These pulmonary surfactants consist of phospholipids and proteins, comprising 90% and 10% of their molecular weight, respectively (99). Four surfactants play crucial roles in the normal regulation and function of pulmonary function: SP-A, SP-B, SP-C, and SP-D. SP-B and SP-C primarily suppress surface tension in alveoli, preventing alveolar collapse and maintaining alveolar health (100). Importantly, SP-A and SP-D, often regarded as collectins, are essential to orchestrate humoral and innate immune responses to various infections and pathogenic stimuli, including bacteria, viruses, and fungi (99). SP-A and SP-D act as opsonization molecules to promote phagocytic clearance of the pathogen upon recognition through several receptors (e.g., SPR-210, complement receptor 3, and calreticulin/CD91) (101–103). Although outside the scope of this review, SP-A and SP-D interact with a variety of other host cell surface receptors on professional phagocytes, as reviewed previously (97, 104). Furthermore, they increase the membrane permeability of pathogens, thereby acting as cytotoxic compounds to enhance the clearance of pathogens (105).

Host SP-A and SP-D promote protective immunity against respiratory fungal infections by inhibiting fungal growth and promoting greater phagocytosis (105). Given the antifungal activity of surfactants, fungal melanin could thwart this arm of the innate immune response, particularly in the initial resting conidia phase. One study demonstrated an affinity for SP-D, but not SP-A, to bind to melanin in resting conidia in a calcium-independent manner and cell wall carbohydrates of germinated conidia in a calcium-dependent manner (106). When SP-D was pre-incubated with melanin, conidia were phagocytosed by macrophages at lower levels compared to conidia exposed to SP-D alone. Further, non-opsonized melanin ghosts resulted in suppressed secretion of pro-inflammatory cytokines, yet the opposite occurred when opsonized with a recombinant human SP-D. These opsonized DHN-melanin ghosts were associated with elevated phagocytosis, inability to quench ROS, and triggered pro-inflammatory cytokine expression and secretion (106). Interestingly, a recent study demonstrated dampened epithelial SP-D mRNA expression when challenged by amelanotic conidia compared to WT conidia (107). These findings illustrate that SP-D plays a crucial role in the initiation of inflammatory responses, therefore showing promising avenues in research as a novel therapeutic. The ability of SP-D to increase clearance of opsonized *A. fumigatus* could be utilized prophylactically in clinical settings that consist of patient populations with higher susceptibility to fungal infections.

Redundancy between each host SP and their ability to effectively opsonize melanized fungal species remains undetermined. No formal investigations have been conducted to explore the role of melanin dependency for SP-mediated clearance of fungal species. There remain no published investigations into interactions between *Rhizopus* and host surfactants. Previous studies examined surfactant interactions with *C. neoformans* through different lenses of capsular and acapsular cell wall components (108). However, these studies did not consider whether melanin alone contributed to surfactant-mediated host responses. Some inferences into melanin-mediated virulence could be extrapolated. For example, the *C. neoformans* polysaccharide capsular component, glucuronoxylomannan, possesses similar immunomodulatory functions as melanin (109). Nevertheless, as *C. neoformans*’ capsular and cell wall make-up vary significantly, formal investigations are warranted. Furthermore, elucidating various SP-binding immune responses to different fungal melanins and the downstream inflammatory responses is of pivotal importance.

### Melanin and oxidative stress

In non-professional phagocytic cells and immune cells, ROS production triggers inflammation and enhances protective responses against invading fungal pathogens. Similar to the regulation of cellular functions by the well-known phosphorylation and dephosphorylation processes, ROS actions in the regulation of cellular signaling are generally mediated by reversible oxidative post-translational modifications within specific target proteins (110). Fungal melanin possesses the remarkable capacity to absorb ROS to serve as an antioxidant for pathogens, including *Aspergillus* spp. (19–22, 35). While the exact mechanisms of how melanin absorbs ROS have not been elucidated, mounting evidence suggests physical interaction between melanin and ROS species (19, 111). Indeed, in *A. fumigatus* conidia lacking melanin, both neutrophils and monocytes generated substantially higher levels of ROS compared to WT conidia with an intact melanin layer (19). Although limited information has been reported for other melanized fungi and their impact on host oxidative stress, *Rhizopus* spp. treated with statins demonstrated reduced melanin production that was associated with greater susceptibility to oxidative stress (30). Furthermore, higher melanin production in *C. neoformans* due to higher pH or lower temperature was associated with improved fungal viability when exposed to oxidative stress (112).

Given the interconnectedness of these soluble mediators, it is likely that melanin targets multiple steps to enhance fungal pathogenesis. For example, calcium-dependent pathways regulate ROS-linked inflammatory phenotypes in lung disease (113–115), and secretion of CXCL8 is highly dependent on ROS production (116). Thus, it is possible that melanin exerts immunosuppressive actions that converge on these important mediators of antifungal defense.

## FUTURE DIRECTIONS

Emerging data highlight the critical role of melanin as a potent virulence factor with the ability to directly modulate host immune functions. Further elucidation of its mechanism of pathogenicity would provide actionable targets for novel antifungals. The identification of a C-type lectin receptor that recognizes DHN-melanin, called MelLec or CLEC1A, reveals one pathway for direct effects in host cells (117). Endothelial and myeloid cells, but not airway epithelial cells, express MelLec (50, 117). As airway epithelial cells are the first point of contact for these three melanized fungal pathogens, it is critical to understand how fungal melanin exerts its effects in airways. Given the lack of MelLec on these cells coupled with the ability of both DHN-melanin and L-DOPA melanin to block epithelial-mediated inflammation, fungal melanin may target other receptor(s) or pathway(s) in these cell types (50). Thus, it urges further investigation to identify other receptors that bind various fungal melanins.

Fungal melanins are polymeric and likely exist in different sizes. During cell wall remodeling, which occurs during the pathogenesis of fungal infections, melanin undergoes remarkable transformation and, in some cases, becomes shed into smaller pieces during conidial swelling and hyphal development. The triggers for increased melanin production and its subsequent release by the organism may differentially impact the host’s immune response. While *C. albicans* produces melanin, the regulation of biosynthesis and its subsequent release into the environment is poorly understood and requires further study. The mechanism(s) by which mammalian cells recognize these fungal melanins of different sizes and compositions remains to be elucidated.

Despite the growing need for therapeutic strategies against fungal infections, there are currently no licensed fungal vaccines available. Additionally, there are only three major classes of antifungal drugs that exist, adding to the complexity of treatment strategy and risk of antifungal resistance (16, 118). Generating new antifungals specifically targeting fungal melanin synthesis, thus rendering them susceptible to immune killing and other antifungals, may be an effective therapeutic strategy. Notably, the administration of glyphosate, a widely used broad-spectrum herbicide, prolonged the survival of mice infected with *C. neoformans* by delaying the melanin synthesis in yeast cells through the inhibition of autopolymerization of L-DOPA as well as the oxidation of L-epinephrine (119). Additionally, *C. neoformans*-infected mice that were immunized with monoclonal antibodies to melanin reduced fungal burden and prolonged survival (120). While this review focused on well-established melanized fungal species commonly associated with respiratory infections, investigations into less common melanized fungal species or those associated through other routes of infection are critical. Interestingly, a melanin-binding antibody developed for fungal research is being tested for targeting melanin in melanoma tumors, illustrating the translational potential of anti-melanin strategies (121). Highlighting this gap, the addition of a specific inhibitor for DHN-melanin biosynthesis, pyroquilon, enhanced the efficiency of caspofungin against *Alternaria infectoria* (122). However, *A. infectoria* grown in the presence of antifungals alone increased its melanin production, which the authors noted could be a potential defense mechanism against antifungals. Regardless, this avenue of investigation warrants additional studies not only for *Aspergillus* spp., *Rhizopus* spp., and *Cryptococcus* spp., but also for less understood melanized fungal pathogens of clinical importance.

## CONCLUSIONS

**Fig 2:**
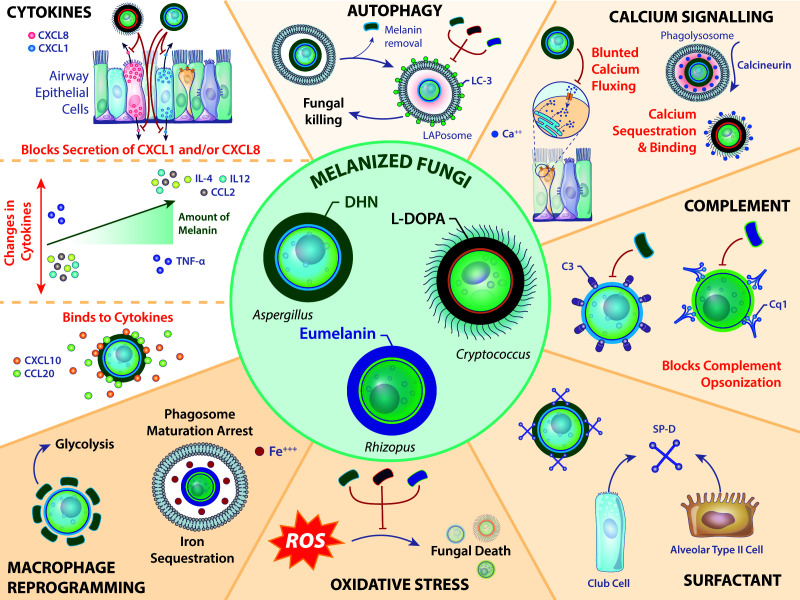
Overview of fungal melanins on host immunity. *Aspergillus*, *Rhizopus*, and *Cryptococcus* are in the center with host immune responses depicted in the periphery. Illustration by Nicole Wolf, MS, ©2025.

Fungal melanins mask the carbohydrates on the fungal cell wall readily recognized by host PRRs. Yet, these melanins serve as more than a passive barrier, as displayed by their impressive immunomodulatory activities to circumvent host antifungal immunity and promote fungal virulence. Both DHN-melanin and L-DOPA exert their effects on host immune cell functions as well as through interactions with soluble mediators (Fig. 2). We need to better understand how these molecular pathways are affected by melanin through indirect and/or direct interactions. Indeed, the use of fungal melanins as a potential therapeutic target may serve as a critical strategy to modulate the immune response in the lung.
